# Perplexity-Based Molecule Ranking and Bias Estimation of Chemical Language Models

**DOI:** 10.1021/acs.jcim.2c00079

**Published:** 2022-02-22

**Authors:** Michael Moret, Francesca Grisoni, Paul Katzberger, Gisbert Schneider

**Affiliations:** † Department of Chemistry and Applied Biosciences, ETH Zurich, RETHINK, Vladimir-Prelog-Weg 4, Zurich 8093, Switzerland; ‡ Institute for Complex Molecular Systems, Department of Biomedical Engineering, Eindhoven University of Technology, Groene Loper 7, Eindhoven 5612AZ, Netherlands; § Center for Living Technologies, Alliance TU/e, WUR, UU, UMC Utrecht, Princetonlaan 6, Utrecht 3584 CB, The Netherlands; ∥ ETH Singapore SEC Ltd., 1 CREATE Way, #06-01 CREATE Tower, Singapore 138602, Singapore

## Abstract

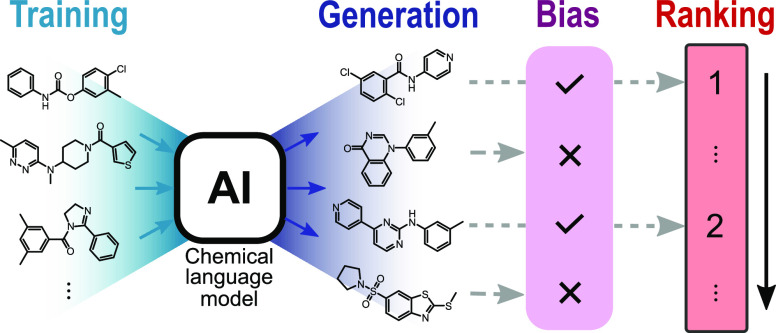

Chemical language models (CLMs) can be employed to design molecules with desired properties. CLMs generate new chemical structures in the form of textual representations, such as the simplified molecular input line entry system (SMILES) strings. However, the quality of these de novo generated molecules is difficult to assess a priori. In this study, we apply the perplexity metric to determine the degree to which the molecules generated by a CLM match the desired design objectives. This model-intrinsic score allows identifying and ranking the most promising molecular designs based on the probabilities learned by the CLM. Using perplexity to compare “greedy” (beam search) with “explorative” (multinomial sampling) methods for SMILES generation, certain advantages of multinomial sampling become apparent. Additionally, perplexity scoring is performed to identify undesired model biases introduced during model training and allows the development of a new ranking system to remove those undesired biases.

## Introduction

**Figure 1:**
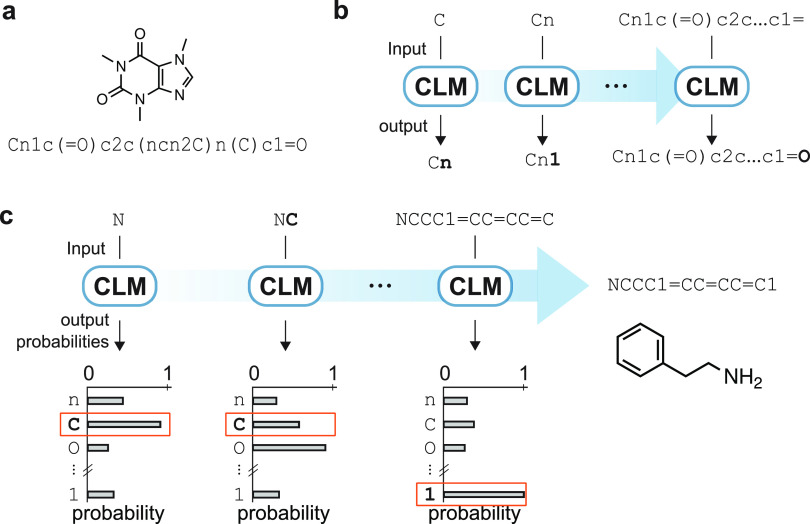
Principles of chemical language models (CLMs). (a) Example of a molecular structure (Kekulé structure) and a corresponding SMILES string. (b) CLMs are trained to iteratively predict the next SMILES character based on the preceding string characters. (c) Multinomial sampling can be used to generate new SMILES strings from trained CLMs, where SMILES characters are sampled with a weighted random sampling of probability distributions learned by the CLM.

Generative deep learning has become a promising method for chemistry and drug discovery.^1−21^ Generative models learn the pattern distribution of the input data and generate new data instances based on learned probabilities.^22^ Among the proposed generative frameworks that have been applied to de novo molecular design,^2−19^ chemical language models (CLMs) have gained attention because of their ability to generate focused virtual chemical libraries and bioactive compounds.^20,21,23^ CLMs are trained on string representations of molecules, *e.g*., simplified molecular input line entry system (SMILES) strings (Figure 1a),^24^ to iteratively predict the next SMILES character using all the preceding portions of the SMILES string (Figure 1b). In this process, CLMs learn the conditional probability of sampling any SMILES character based on the preceding characters in the string. After training, the model can be used for molecular construction. CLMs have been demonstrated to both learn the SMILES syntax and implicitly capture “semantic” features of the training molecules, such as physicochemical properties,^20,21,25,26^ bioactivity,^2,21^ and chemical synthesizability.^3^ Although alternative generative approaches have been proposed for de novo design,^13,27−29^ benchmarks have not shown these to outperform CLMs.^30,31^ A feature of CLMs is their ability to function in low-data regimes,^25,29^*i.e.*, with limited training data (typically in the range of 5–40 molecules).^2,3,25^ One of the most widely employed approaches for low-data model training is transfer learning.^20,32^ This method leverages previously acquired information on a related task for which more data are available (″pretraining”) before training the CLM on a more specific limited dataset (″fine-tuning”).^33^

Several prospective de novo design studies based on CLMs used weighted random sampling (*i.e.*, multinomial sampling, often in the form of temperature sampling) for molecule generation.^2,20,25,34^ This method samples the most likely SMILES string characters more frequently than the unlikely characters. This feature enables (i) extensive virtual molecule libraries to be generated and (ii) a certain chemical space to be investigated owing to “fuzzy” (probability-weighted) random sampling. However, such a sampling strategy can result in molecules that do not possess the physicochemical and biological properties of the training data. Furthermore, because the number of molecules that can potentially be sampled from CLMs considerably exceeds synthetic capacities, and a natural ranking of the generated SMILES does not exist, an additional procedure is required for molecule prioritization, *e.g*., one based on similarity assessment or activity prediction.^2,35^ We recently introduced the beam search algorithm as an alternative to multinomial sampling. During beam search, the most likely SMILES strings are generated based on the respective character probabilities, thereby alleviating the strict requirement for additional molecule prioritization.^36^ The beam search performs chemical space “exploitation” as the algorithm searches for the most probable SMILES strings in a greedy manner. This method generates only a few candidate molecules at the expense of chemical space exploration and design diversity.

Herein, we aimed to improve upon an existing CLM that we recently used for prospective application^3,25,36^ to increase its potential for automated molecular design and scoring. To this end, we used perplexity to assess the “goodness” of the designs generated by CLMs via multinomial sampling aiming to (i) preserve the advantage of intrinsic molecule ranking^37^ as it can be achieved via beam search, (ii) benefit from the chemical space exploration provided by multinomial sampling, and (iii) provide model-based insights into the most promising molecules for follow-up analysis.^36^ We systematically trained a CLM on sets of bioactive ligands of 10 different macromolecular targets under four different data-regime scenarios each. On top of its ability to rank the generated SMILES strings, the perplexity metric identified undesired effects of transfer learning using CLMs, thereby qualifying as a criterion for detecting undesired model bias.

## Results and Discussion

### Perplexity-Based Scoring of Molecular De Novo Designs

This study aimed to leverage the overall likeness of the generated SMILES strings for automated molecule ranking based on the respective character probabilities. Accordingly, the SMILES string(s) with the highest likeness can be considered the best-matching solutions to the CLM sampling problem, reflecting the information learnt by the CLM. We selected the perplexity metric to reflect the probability of sampling a SMILES string as a function of its characters (eq 1).^37^ Perplexity has been used to assess the performance of language models in natural language processing.^37−39^ For a SMILES string of length *N*, the perplexity score can be computed by considering the CLM probability of any *i*th character (*p_i_*):
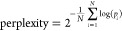
1

The information on the overall character probabilities is captured into a single metric, which is normalized by the length of the SMILES string (*N*). Perplexity allows quantifying the CLM confidence that a specific SMILES string could have belonged to the training data. If the assumption that the underlying CLM captured relevant information from the training data is satisfied, then perplexity will be suitable for molecule ranking. Because the training objectives of the CLM are implicitly encoded in the fine-tuning data, the perplexity score allows one to assess whether the generated SMILES strings match the objectives. A SMILES string composed of probable characters (high *p_i_* values) exhibits low perplexity, whereas a string containing many unlikely characters (low *p_i_* values) exhibits high perplexity. Hence, low perplexity scores are desirable.

To analyze the behavior of perplexity, an RNN with long short-term memory (LSTM) cells^40^ was pretrained with approximately 1.6 million molecules from ChEMBL (version 28).^41^ Ten randomly selected targets were used for fine-tuning (Table 1). For each macromolecular target, ligands that possessed a pChEMBL activity value larger than 6 were selected, where pChEMBL is defined as −log_10_(molar IC_50_, XC_50_, EC_50_, AC_50_, *K_i_*, *K*_d_, or potency). To emulate different low-data regimes typical of drug discovery, we prepared fine-tuning sets of different sizes that contained 5, 10, 20, or 40 randomly selected ligands for each target. For each of the 10 targets and each of the four fine-tuning sets, a total of 1000 SMILES strings were sampled after every second epoch during a total of 100 CLM fine-tuning epochs via multinomial sampling. Unlike our previous studies,^2,3,25,35^ no temperature parameter was used to modify multinomial sampling to avoid the introduction of confounding factors in our analysis. For all fine-tuned models and all the fine-tuning epochs, the mean SMILES string validity consistently exceeded 90% (Figure S1).

**Table 1:** Macromolecular Targets Selected for CLM Fine-Tuning^a^

CHEMBL ID	target	protein classification
CHEMBL1836	prostanoid EP4 receptor	G protein-coupled receptor
CHEMBL1945	melatonin receptor 1A	G protein-coupled receptor
CHEMBL1983	serotonin 1D (5-HT_1D_) receptor	family A G protein-coupled receptor
CHEMBL202	dihydrofolate reductase	oxidoreductase
CHEMBL3522	cytochrome P450 17A1	cytochrome P450
CHEMBL4029	interleukin-8 receptor A	family A G protein-coupled receptor
CHEMBL5073	CaM kinase I delta	kinase
CHEMBL5137	metabotropic glutamate receptor 2	G protein-coupled receptor
CHEMBL5408	serine/threonine-protein kinase TBK1	kinase
CHEMBL5608	NT-3 growth factor receptor	kinase

a ChEMBL target identifier, generic target name, and protein classification based on the respective ChEMBL target report card.

### Chemical Relevance of Perplexity

To investigate the information captured by the perplexity metric, we evaluated its correlation with two measures of molecular similarity computed between the de novo designs and the corresponding fine-tuning sets: (a) Tanimoto similarity on Morgan fingerprints,^42^ which captures the presence of common substructures, and (b) Tanimoto similarity on topological pharmacophore fingerprints,^43^ which captures the presence of shared structural motifs relevant for ligand-target interactions. To allow for an easier comparison with the perplexity metric, the computed Tanimoto similarity values were converted into distances (computed as 1 – similarity).

**Figure 2:**
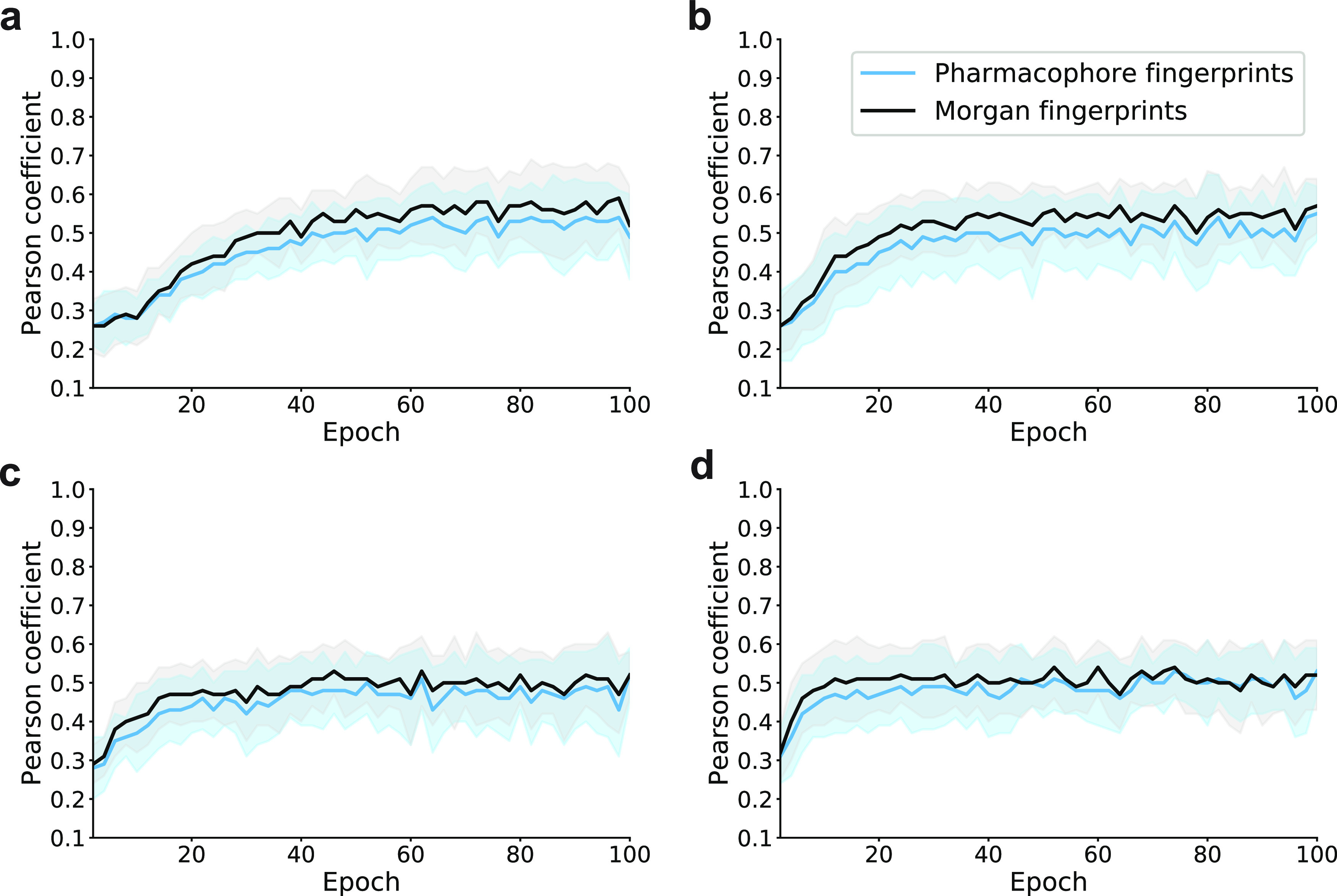
Correlation between the perplexity score and other representations. Pearson correlation coefficient between the perplexity score and the average distance of the generated molecules during fine-tuning (black line, Morgan fingerprints; blue line, 2D pharmacophore fingerprints). Gray shaded areas indicate standard deviations. Fine-tuning sets contained (a) 5, (b) 10, (c) 20, and (d) 40 molecules.

A Pearson correlation of approximately 0.3 was observed between the perplexity score and the computed distances to the smaller fine-tuning sets (5 and 10 molecules) during the initial fine-tuning epochs before stabilizing at a value of 0.5 (Figure 2). A correlation of 0.5 tends to be reached at earlier epochs with bigger fine-tuning sets compared to using only five molecules (Figure 2). This result suggests that the perplexity score captures common substructure and 2D pharmacophore features while at the same time incorporating additional information modeled by the CLM.

### Perplexity as an Indicator for Comparing Molecular Sampling Strategies

In a previous study, new bioactive compounds were successfully identified via beam search sampling,^36^ which is a heuristic greedy algorithm. Its search ″breadth″ is controlled by a width parameter (*k*), which represents the number of the most probable SMILES strings that the model considers during string extension. Here, beam search was used as a reference method for comparison with multinomial sampling.

We investigated the difference in perplexity scores between molecules generated using a CLM via either beam search or multinomial sampling. To this end, we additionally sampled for each of the 10 targets and each of the four fine-tuning sets, 10 or 50 SMILES strings via beam search sampling (*k* = 10 or 50).

**Figure 3:**
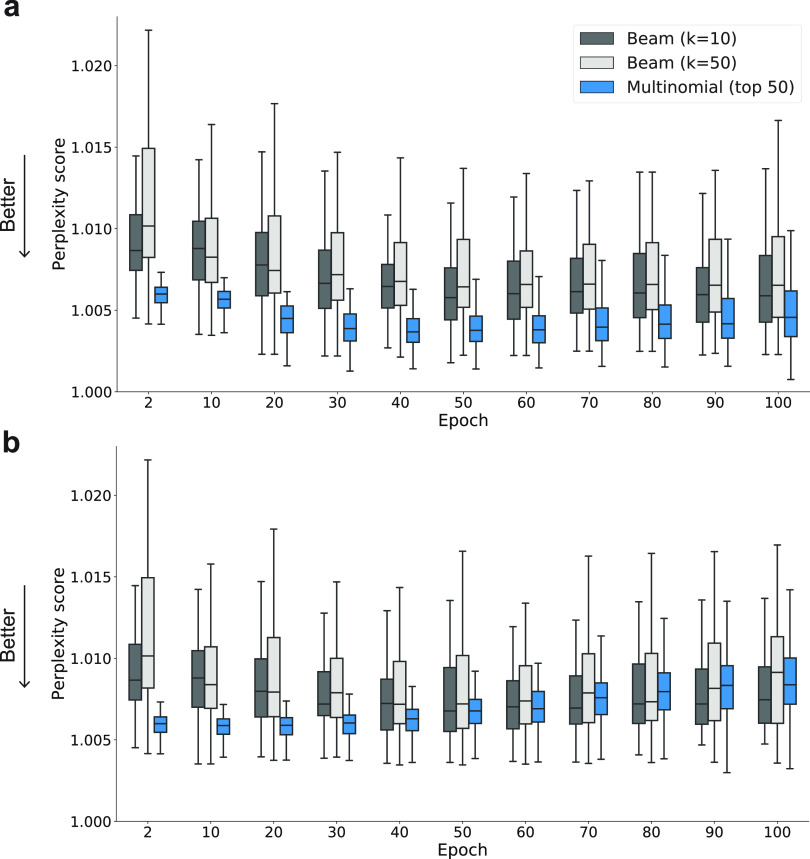
Variation in perplexity during fine-tuning. (a) Distribution of the top-scoring compounds for each method over 100 fine-tuning epochs (only every 10 epochs shown in the graph for clarity). (b) Distribution of the top-scoring compounds by considering only molecules with a similarity below 50% (Tanimoto index computed on Morgan fingerprints) to the closest molecule in their respective fine-tuning set. Median and lower to upper quartile values reported using boxplots for 10 different protein-specific fine-tuning sets, which contain five molecules each. Boxplots for fine-tuning sets with sizes of 10, 20, and 40 molecules are provided in the Supporting Information. Arrows indicate the direction of optimal perplexity values (“Better”).

In CLM fine-tuning using the smallest fine-tuning sets (five molecules), multinomial sampling consistently outperformed beam search sampling in terms of the perplexity score as molecules with the best score (lowest perplexity) were obtained (Figure 3a). Increasing the beam search width from *k* = 10 to 50 did not markedly improve the ability of this method in identifying molecules with higher perplexity scores (Figure 3). These observations were confirmed for the larger fine-tuning sets (Figures S2–S4). A potential explanation for this observation is the “greedy” nature of the beam search,^44^ which explores only a limited number of possibilities for next-character addition. By contrast, the “fuzzy” nature of multinomial sampling allows the generation of a greater number of molecules and hence a broader exploration of the chemical space of interest.

The 50 top-scoring molecules generated via multinomial sampling not only indicated lower median perplexity values but also spanned a narrower range of values (Figure 3a). This suggests that multinomial sampling yields a greater number of high-scoring designs (low perplexity) for follow-up synthesis and biological testing than the beam search algorithm (Figure 3a and Figures S2–S4).

When filtering out designs with a substructure similarity (Tanimoto index on Morgan fingerprints^42^) greater than 50% of the respective fine-tuning molecules, the difference between multinomial sampling and beam searching was less pronounced (Figure 3 and Figures S5–S7). Multinomial sampling identified molecules with lower perplexity scores than the beam search in 72% of the cases involving the smallest fine-tuning sets (Table S1). The deterioration in performance for highly diverse molecules was less pronounced with the larger fine-tuning sets (Table S1).

The results of this study corroborate the potential of multinomial sampling, not only for chemical space exploration to obtain chemically diverse molecular designs but also for generating high-scoring compounds that are sufficiently diverse from the fine-tuning compounds.

### Assessing Pretraining Bias Based on Perplexity

CLM pretraining might impose a greater effect on model performance than CLM fine-tuning as model pretraining is typically performed with data that are at least 2 orders of magnitude higher in amount than fine-tuning.^2,3,25,35,36^ If a molecule is generated by a CLM due to pretraining only, then it will not necessarily match the design objectives as represented by the fine-tuning data. We analyzed the degree to which new molecules were generated due to the sole effect of pretraining, *i.e.*, we verified whether “pretraining bias” occurred. In principle, for a CLM, perplexity can be used to score any molecule, including those that are not generated by the model. This can be achieved by computing the conditional probabilities of each SMILES character using the CLM. Therefore, the perplexity score was employed to differentiate between the information learned by the CLM during pretraining and during fine-tuning to score the molecules generated at a specified fine-tuning epoch. First, for each fine-tuning epoch, molecules were scored and ranked by the perplexity of the model used to generate them. Subsequently, each de novo design was scored and ranked based on the perplexity of the CLM after pretraining (*i.e.*, prior to any fine-tuning). We hypothesized that a suitable ranking method should favor molecules that were generated based on information learned by the model during fine-tuning (capturing the final objectives of the experiment) and downrank the molecules generated based solely on pretraining (capturing “generic” information).

To seize this concept quantitatively, we subtracted the rank yielded by the pretrained model (rank_pt_) from that of the fine-tuned CLM (rank_ft_) for each molecule and defined this difference as the “delta” score (eq 2), as follows:
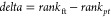
2

Molecules with a positive delta score were considered more likely to be output by the fine-tuned CLM. A negative delta score suggests that the fine-tuning procedure renders a certain molecule less likely to be output than after pretraining, which does not satisfy the design objectives. Thus, when a molecule possesses a good rank based on the perplexity after fine-tuning but it has a negative delta value, it should not be considered for further experiments.

**Figure 4:**
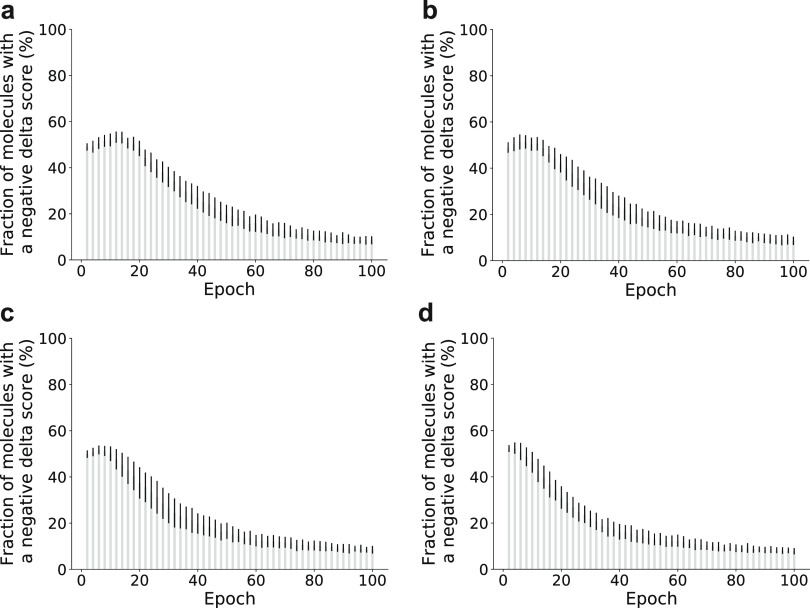
Delta score during fine-tuning experiments in a low-data regime. Percentage of molecules with a negative delta score (1000 sampled SMILES strings; mean ± standard deviation reported for 10 different target proteins). Fine-tuning sets of (a) 5, (b) 10, (c) 20, and (d) 40 molecules.

The delta score was computed for all sampled molecules during fine-tuning experiments (Figure 4). The percentage of molecules with a negative delta score exceeded 40% for the first 20 fine-tuning epochs and remained above 10% until the end of fine-tuning for all fine-tuning set sizes. This observation suggests that 10–40% of the molecules were generated based on “generic” pretraining, instead of “task-focused” fine-tuning. As such, this outcome highlights the practicability of the proposed delta score as an indicator to (i) detect potential pretraining bias, (ii) identify the best-suited epoch for a productive sampling of molecules that fulfill the study goals, and (iii) select the most promising de novo designs.

To expand the analysis, we focused on the 50 top-scoring molecules generated via multinomial sampling. We discovered that, among them, only up to 3% of the molecules received a negative delta score (Figure S8). This shows that using perplexity alone reduces the pretraining bias. However, the pretraining bias was not completely removed, which highlights the benefits of using both the perplexity and delta score for molecule prioritization prior to synthesis and biological testing.

In summary, these results suggest that the potential of generative CLMs in medicinal chemistry can be expanded by employing the SMILES perplexity for molecule prioritization and for detecting potential pretraining bias.

## Conclusions

This present study constitutes a step forward toward an automated, self-supervised de novo design. By serving as a model-intrinsic score, perplexity enables the quality assessment of generated molecules. In particular, perplexity might be useful for identifying the most promising molecules, *i.e.*, those that match the probability distribution of the training data as captured by the CLM. This approach enabled the comparison of two different methods for SMILES sampling from a trained CLM. The results revealed certain advantages of multinomial sampling over the beam search method for molecule generation. Because perplexity can be used to score SMILES strings based on the information learned by a CLM, the pretraining bias can be identified based on the newly introduced delta score. Perplexity combined with the delta score can reveal the most promising molecules, in terms of the fine-tuning objectives, for synthesis and testing. These features can further accelerate drug discovery using CLMs. Future studies will focus on the combination of perplexity with the temperature parameter of multinomial sampling or SMILES augmentation.^34,45^ Furthermore, the combination of CLMs and perplexity scoring bears promise for screening large collections of commercially available compounds to accelerate model validation.^46^ More experiments should be performed to determine the effect of the new approach on molecular de novo designs involving CLMs.

## Data and Software Availability

The computational framework presented herein, pretrained neural network weights, and data used for model training are available in a GitHub repository from URL https://github.com/ETHmodlab/CLM_perplexity.

## Methods

### Data Processing

Molecules were represented as canonical SMILES strings using an RDKit (2019.03.2). SMILES strings were standardized in Python (v3.6.5) by removing salts and duplicates, and only SMILES strings with 20–90 characters were retained.

### Pretraining Set

The molecules were retrieved from ChEMBL28.^47^ After data processing, the pretraining dataset contained 1,683,181 molecules encoded as SMILES strings. This set was further segregated randomly into a training set (1,599,021 molecules) and a validation set (84,160 molecules).

### Fine-Tuning Sets

Target selection was limited to molecules satisfying the following conditions (ChEMBL annotation): (i) organism: *Homo sapiens*; (ii) protein classification (L1): enzymes, membranes receptors, transcription factors, and single proteins; (iii) number of compounds: 962–2057 molecules (range defined by ChEMBL); (iv) number of activities: at least 2000 reported pChEMBL values. Ten target proteins were randomly selected from the list of targets. For each of the 10 selected target proteins, sets of 5, 10, 20, and 40 molecules with pChEMBL >6 were compiled randomly.

### CLM Implementation and Training

All computational experiments were implemented in Python (v3.6.5) using Keras (v2.2.0, https://keras.io/) with the Tensorflow GPU backend (v1.9.0, https://www.tensorflow.org/). CLMs were implemented using a recurrent neural network with long short-term memory cells (LSTM).^40^ The network, which was composed of four layers comprising 5,820,515 parameters (layer 1: batch normalization; layer 2: LSTM with 1024 units; layer 3: LSTM with 256 units; layer 4: batch normalization), was trained with SMILES strings encoded as one-hot vectors. We used the Adam optimizer with a learning rate of 10^–4^ for the CLM training during 90 epochs,^48^ where one epoch was defined as one pass over all the training data. Fine-tuning was performed by further training the CLM on the fine-tuning set for 100 epochs.

### Multinomial Sampling

Multinomial sampling was performed based on the CLM output for each SMILES string character. In particular, the probability of each *i*th character to be sampled (*p_i_*) was computed using eq 3:
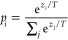
3where *z_i_* is the CLM output for the *i*th character (before applying the softmax function) and *j* runs over all the characters of the dictionary. *T* represents the temperature parameter, which in this study was set to *T* = 1. An analysis of the effect of *T* on the SMILES generation process can be found elsewhere.^25^

### Beam Search Sampling

We use the implementation provided in ref^36^ (https://github.com/ETHmodlab/molecular_design_with_beam_search) using two different beam widths (*k* = 10 and 50).

### Molecular Fingerprints

All fingerprints were computed with the RDKit (2019.03.2) with default settings: (a) Morgan fingerprints were used with a radius of 2 and length of 1024; (b) 2D pharmacophore fingerprints were used with the pre-configured signature factory as published by Gobbi and Poppinger.^43^

## Supporting Information Available

Table S1. Comparison of multinomial sampling and beam search for identifying high-scoring molecules, Figure S1. Validity of the sampled SMILES strings during fine-tuning, Figure S2. Variation of the perplexity score during fine-tuning (10 fine-tuning molecules), Figure S3. Variation of the perplexity score during fine-tuning (20 fine-tuning molecules), Figure S4. Variation of the perplexity score during fine-tuning (40 fine-tuning molecules), Figure S5. Variation of the perplexity score during fine-tuning (10 fine-tuning molecules; threshold on similarity), Figure S6. Variation of the perplexity score during fine-tuning (20 fine-tuning molecules; threshold on similarity), Figure S7. Variation of the perplexity score during fine-tuning (40 fine-tuning molecules; threshold on similarity), and Figure S8. Variation of the delta score during fine-tuning (50 top-ranked molecules) (PDF)
