# Supplementary material for: Perplexity-Based Molecule Ranking and Bias Estimation of Chemical Language Models
Source: J Chem Inf Model. 2022 Feb 22;62(5):1199–206. doi: 10.1021/acs.jcim.2c00079 (PMC8924923; doi:10.1021/acs.jcim.2c00079)
Supplement: Supplementary file 1 — ci2c00079_si_001.pdf [file ci2c00079_si_001.pdf]

SUPPORTING INFORMATION

Perplexity-based Molecule Ranking and Bias Estimation of Chemical Language Models

Michael Moret,[1+] Francesca Grisoni, [2,3+]* Paul Katzberger,[1] Gisbert Schneider[1,4]*

[1]ETH Zurich, Department of Chemistry and Applied Biosciences, RETHINK, Vladimir-Prelog-Weg 4, 8093 Zurich, Switzerland.

[2]Eindhoven University of Technology, Institute for Complex Molecular Systems, Department of Biomedical Engineering, Groene Loper 7, 5612AZ Eindhoven, Netherlandsl

[3]Center for Living Technologies, Alliance TU/e, WUR, UU, UMC Utrecht, Princetonlaan 6, 3584 CB Utrecht, The Netherlands.

[4]ETH Singapore SEC Ltd, 1 CREATE Way, #06-01 CREATE Tower, Singapore 138602, Singapore.

*Correspondence to Gisbert Schneider (gisbert@ethz.ch) and Francesca Grisoni (f.grisoni@tue.nl).

[+]M.M. and F.G. contributed equally to this work.

Table S1 | Comparison of multinomial sampling and beam search for identifying high-scoring molecules. *N* represents the number of times the highest-scoring molecule (based on the perplexity score) was identified by the method, divided by the maximum possible value. "*N* (distance > 0.5)" considers only molecules with 50% maximum similarity to the closest molecule in the respective fine-tuning set (Tanimoto similarity on Morgan fingerprints).

Methods	Fine-tuning sets size	*N*	*N* (distance > 0.5)
Multinomial	5	50/50	36/50
	10	50/50	44/50
	20	50/50	41/50
	40	49/50	50/50
Beam (k = 10)	5	0/50	1/50
	10	0/50	7/50
	20	2/50	7/50
	40	2/50	0/50
Beam (k = 50)	5	0/50	13/50
	10	0/50	7/50
	20	4/50	9/50
	40	2/50	0/50

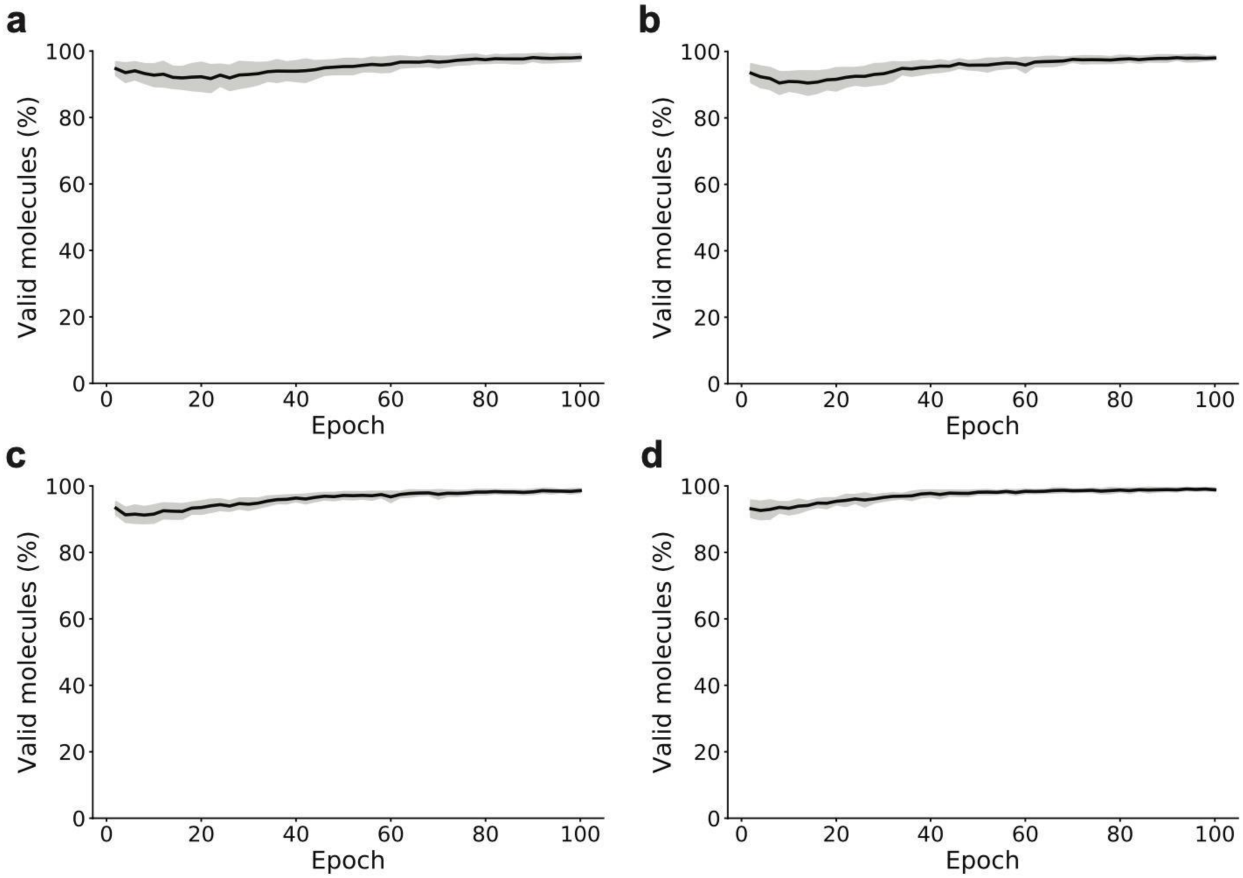

Fig. S1 | Validity of the sampled SMILES strings during fine-tuning. Percentage of valid SMILES strings out of the 1,000 sampled SMILES strings sampled by epoch (mean ± standard deviation reported across 10 different target proteins). Fine-tuning sets with **a,** 5, **b,** 10, **c,** 20, and **d,** 40 molecules.

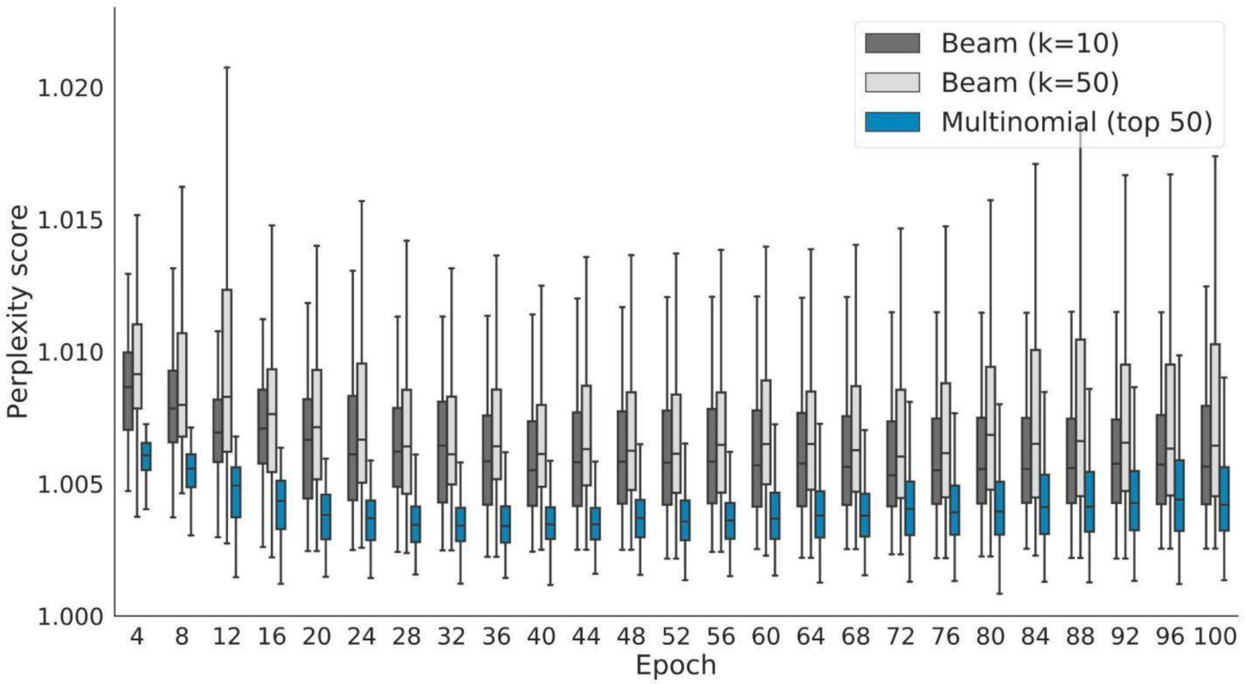

Fig. S2 | Variation of the perplexity score during fine-tuning (10 fine-tuning molecules). Distribution of top-scoring compounds by each method for 100 transfer learning epochs. Median and percentiles are reported for 10 different fine-tuning sets, which contain 10 molecules each, corresponding to 10 different protein targets.

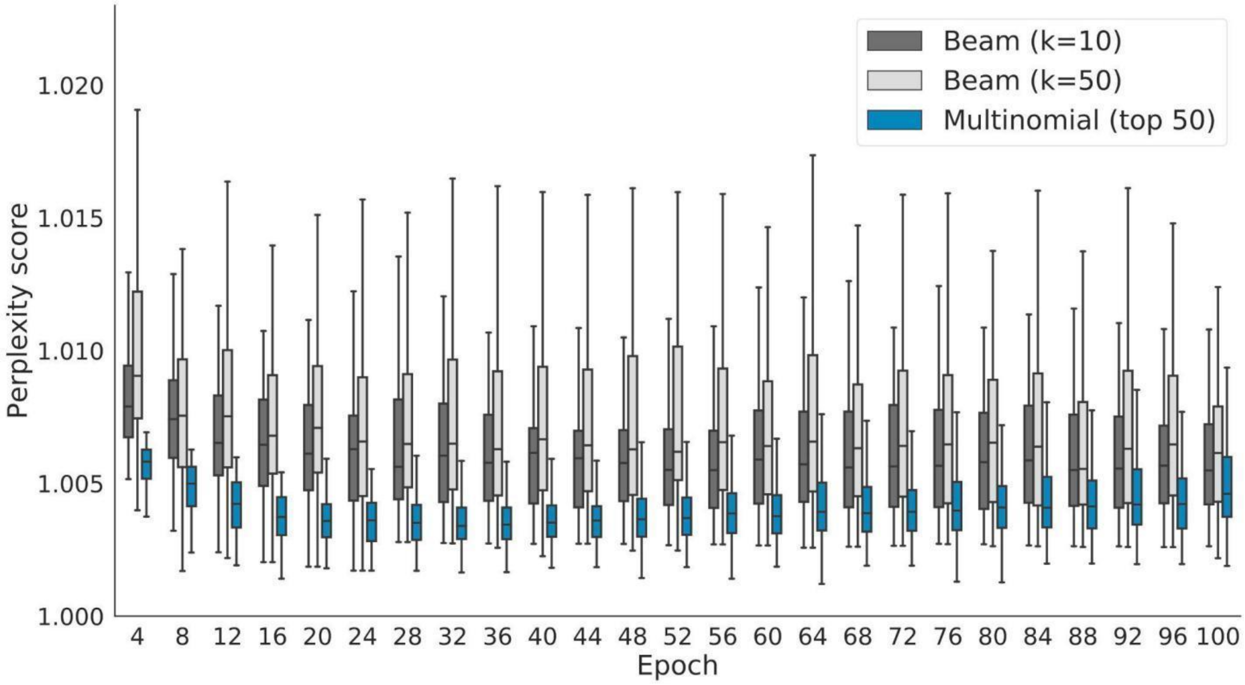

Fig. S3 | Variation of the perplexity score during fine-tuning (20 fine-tuning molecules). Distribution of top-scoring compounds by each method for 100 transfer learning epochs. Median

and percentiles are reported for 10 different fine-tuning sets, which contain 20 molecules each, corresponding to 10 different protein targets.

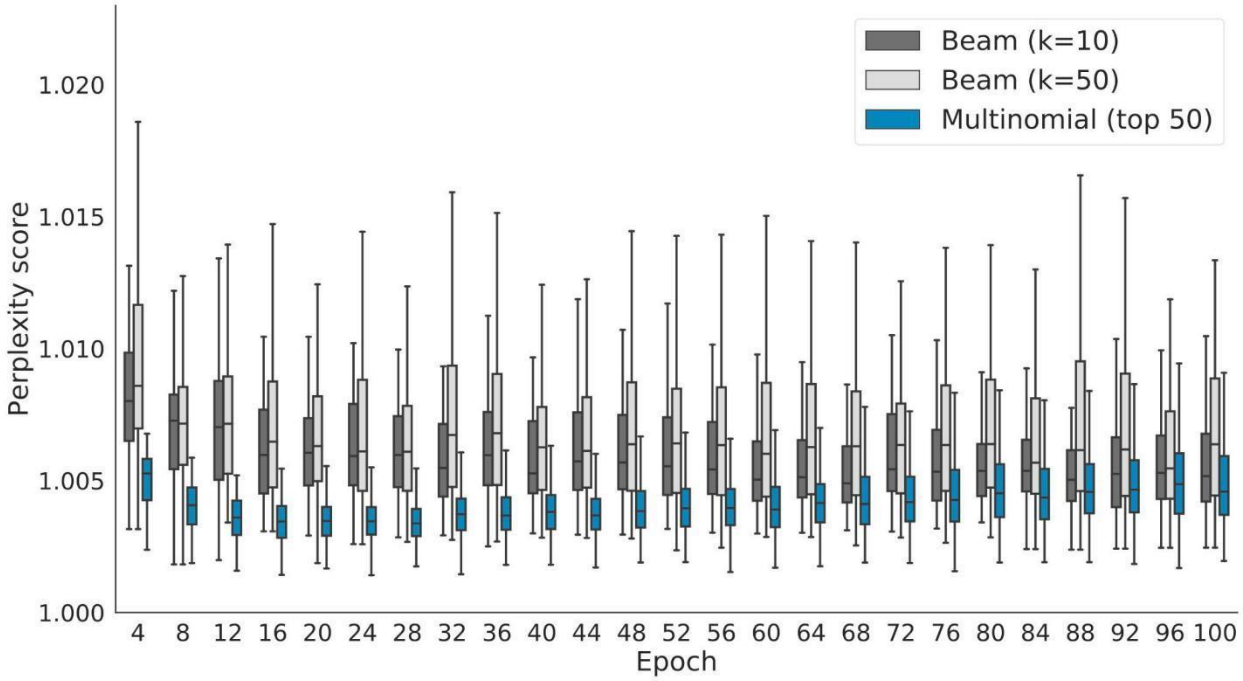

Fig. S4 | Variation of the perplexity score during fine-tuning (40 fine-tuning molecules). Distribution of top-scoring compounds by each method for 100 transfer learning epochs. Median and percentiles are reported for 10 different fine-tuning sets, which contain 40 molecules each, corresponding to 10 different protein targets.

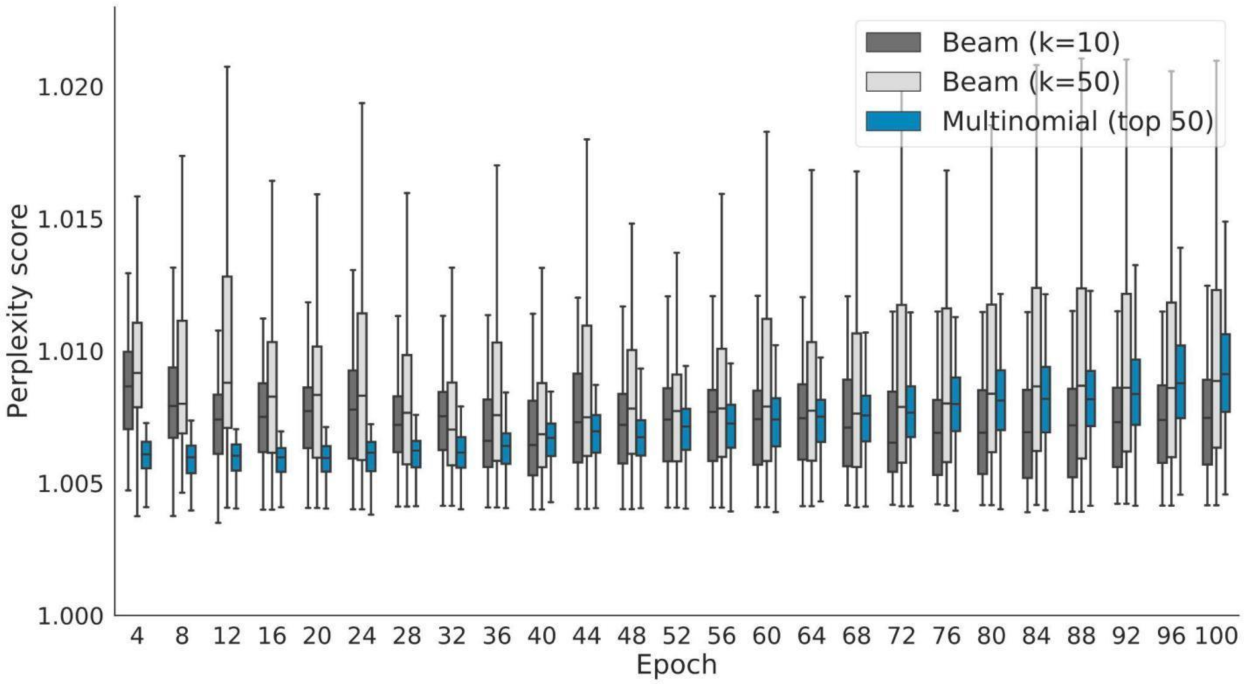

Fig. S5 | Variation in perplexity during fine-tuning (10 fine-tuning molecules; threshold on similarity). Distribution of top-scoring compounds with maximum similarity threshold to fine-tuning set molecules of 50% based on Tanimoto similarity computed using Morgan fingerprints. Median and percentiles are reported for 10 different fine-tuning sets, which contain 10 molecules each, corresponding to 10 different protein targets.

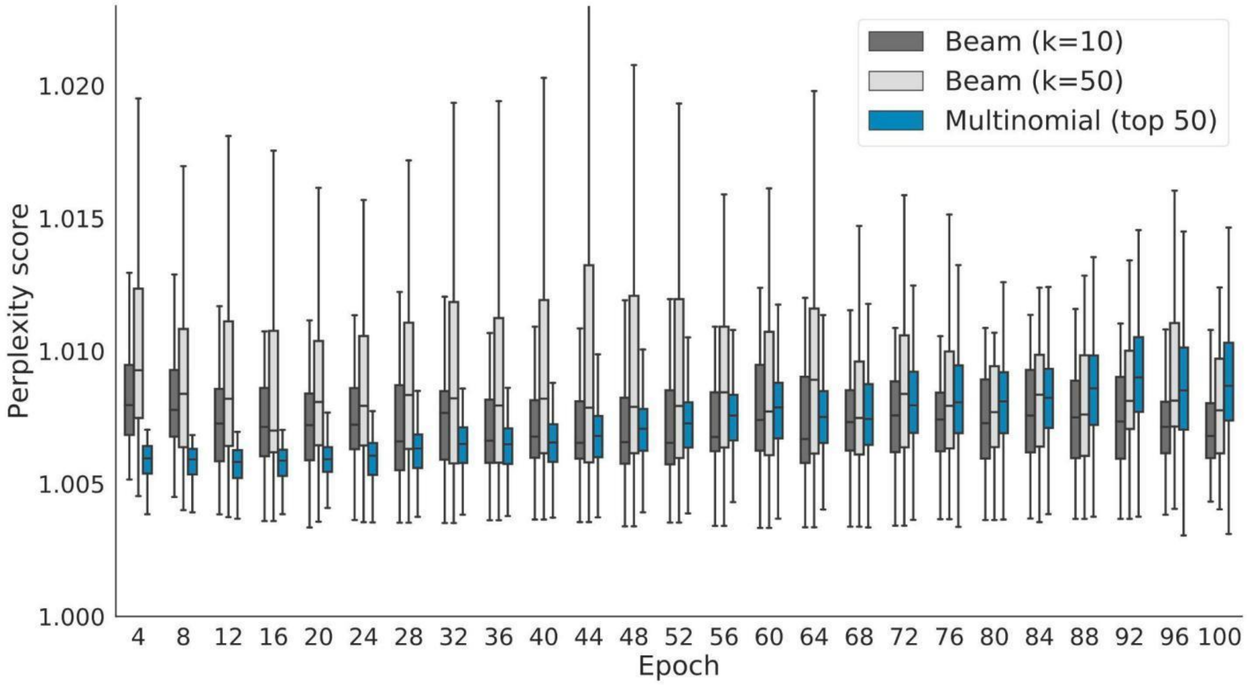

Fig. S6 | Variation in perplexity during fine-tuning (20 fine-tuning molecules; threshold on similarity). Distribution of top-scoring compounds considering only molecules with similarity less

than 50% (Tanimoto index computed using Morgan fingerprints) to the closest molecule in their respective fine-tuning set. Median and percentiles are reported for 10 different fine-tuning sets, which contain 20 molecules each, corresponding to 10 different protein targets.

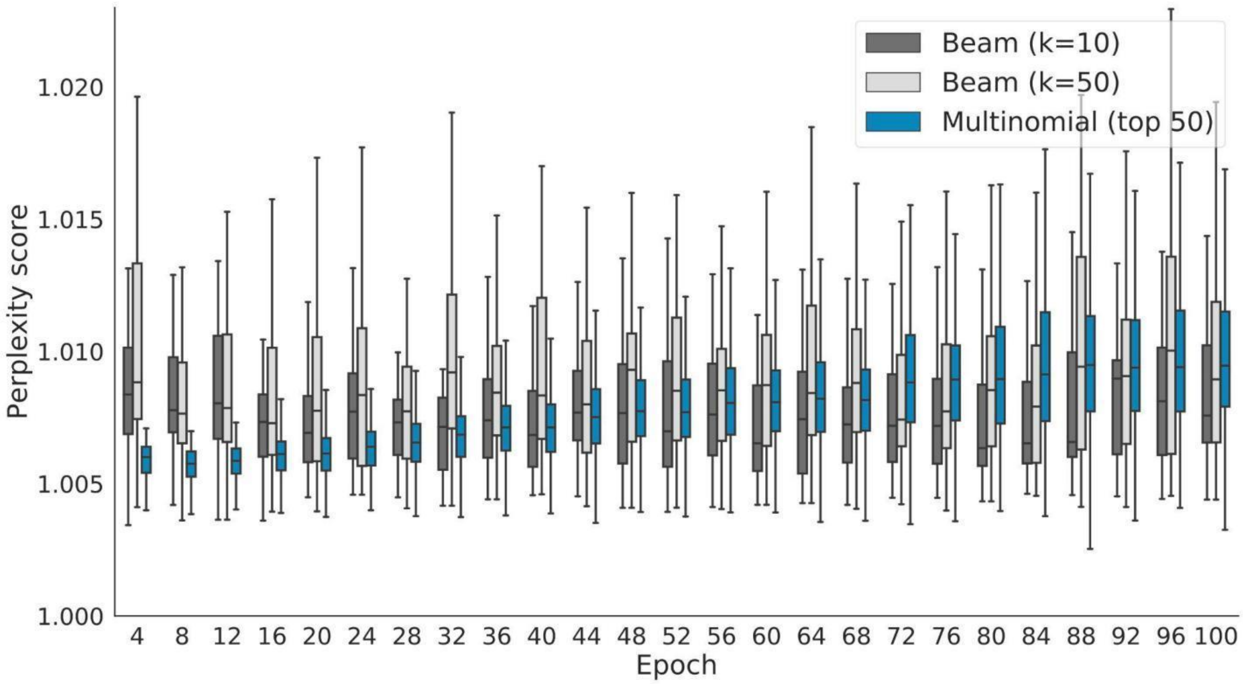

Fig. S7 | Variation in perplexity during fine-tuning (40 fine-tuning molecules; threshold on similarity). Distribution of top-scoring compounds considering only molecules with similarity less than 50% (Tanimoto index computed using Morgan fingerprints) to the closest molecule in their respective fine-tuning set. Median and percentiles are reported for 10 different fine-tuning sets, which contain 40 molecules each, corresponding to 10 different protein targets.

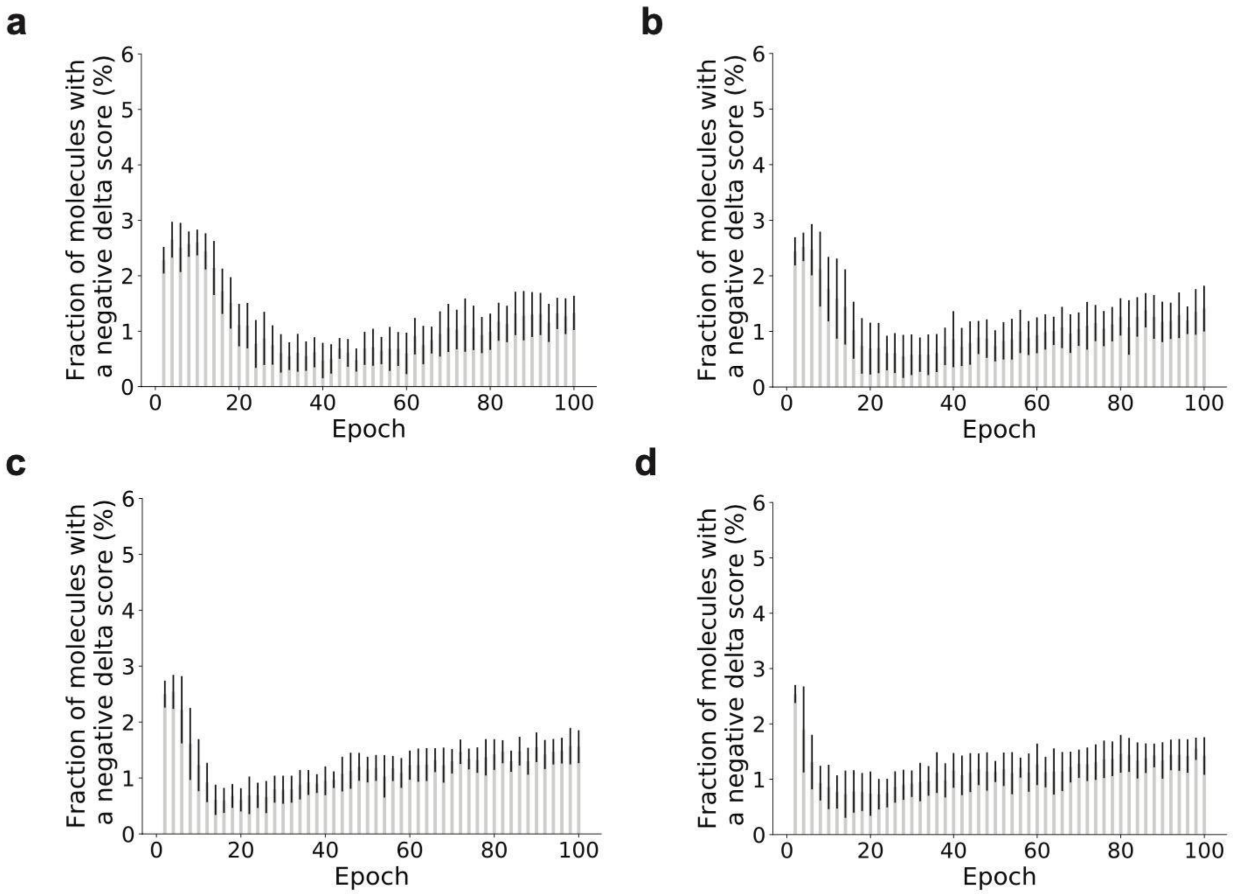

Fig. S8 | Variation of Delta score during fine-tuning (50 top-ranked molecules). Percentage of molecules with negative Delta in focused virtual chemical libraries created from 1,000 sampled SMILES strings (mean ± standard deviation reported across 10 different target proteins). Fine-tuning sets with **a,** 5, **b,** 10, **c,** 20, and **d,** 40 molecules.
